# Severe and refractory hypocalcaemia secondary to osteoblastic bone metastases in bladder signet ring carcinoma: A case report and literature review

**DOI:** 10.1097/MD.0000000000029731

**Published:** 2022-06-30

**Authors:** Wanling Zeng, Du Soon Swee

**Affiliations:** a Department of Endocrinology, Singapore General Hospital, Singapore.

**Keywords:** denosumab, osteoblastic bone metastases, refractory hypocalcaemia, signet ring bladder cancer

## Abstract

Symptomatic hypocalcaemia is uncommon, occurring in <2% of patients with malignancy. Osteoblastic bone metastasis as a cause of hypocalcaemia is rare and not reported in bladder cancer.

**Patient concerns:**

We report a case of refractory hypocalcaemia in a patient with bladder cancer with extensive osteoblastic bone metastases. A 64-year-old male with a history of signet ring bladder carcinoma with osteoblastic bone metastases presented with severe hypocalcaemia with corrected calcium of 1.64 (2.09–2.46) mmol/L as well as hypomagnesemia and hypophosphatemia. He was previously treated with chemotherapy and immunotherapy. Denosumab was also initiated for the prevention of skeletal-related events.

**Diagnoses:**

Additional investigations showed significantly elevated bone formation markers N-terminal propeptide of type I procollagen and alkaline phosphatase. Chest radiography and computed tomography scan also demonstrated extensive areas of sclerotic bone lesions suggestive of osteoblastic bone metastases. He was diagnosed with severe hypocalcaemia secondary to osteoblastic bone metastases and partly to denosumab, vitamin D deficiency, and hypomagnesemia.

**Interventions:**

He was treated aggressively with calcium and vitamin D replacement.

**Outcomes:**

Despite prolonged intravenous calcium replacement and high doses of oral calcium, cholecalciferol, and calcitriol replacement, he had persistent hypocalcaemia with calcium levels ranging from 1.8 to 1.9 mmol/L. He died 4 months after his admission.

**Lessons:**

Osteoblastic bone metastases lead to an increased influx of calcium and phosphate into the bone leading to hypocalcaemia and should be considered as a differential in severe and refractory hypocalcaemia. It is rare and has not been described in bladder cancer. Precaution should be taken upon the initiation of antiresorptive in patients with osteoblastic bone metastases.

## 1. Introduction

Symptomatic hypocalcaemia, an uncommonly encountered complication of malignancy, was reported in about 1.6% of patients admitted under oncology.^[1]^ The pathophysiology of hypocalcaemia in affected patients is often multifactorial, of which osteoblastic bone metastasis is a rare cause. Bone metastasis can be classified as osteolytic, osteoblastic, or mixed and can lead to dysregulation of bone metabolism. Osteoblastic bone metastasis contributing to hypocalcaemia is typically described in prostate and breast cancer^[2,3]^ and rarely in gastric and salivary carcinoma.^[4,5]^ To date, hypocalcaemia resulting from osteoblastic bone metastases in bladder cancer has not been reported. Bone is a common site of metastasis and can occur in 40% of bladder cancer.^[6]^ Signet ring cell carcinoma of the bladder is a rare and aggressive variant of adenocarcinoma. We report a case of refractory severe hypocalcaemia secondary to osteoblastic bone metastases in a patient with metastatic signet ring cell carcinoma of the bladder. In view of the paucity of published information, a review of literature on the cases of osteoblastic bone metastases induced hypocalcaemia to further characterise this rare condition is also presented.

## 2. Case presentation

A 64-year-old male initially admitted for ex-percutaneous nephrostomy site leakage was referred to our department for severe hypocalcaemia with corrected nonionized calcium of 1.64 mmol/L. He had a significant oncological history of metastatic signet ring bladder cancer diagnosed 1 year ago with rapid disease progression despite systemic treatment with chemotherapy and immunotherapy. At diagnosis, he was started on subcutaneous denosumab (Xgeva) 120 mg every 3 months for the prevention of skeletal-related events (SREs) in view of his extensive bone metastases in the sacrum and pelvis. He had received 3 doses of denosumab in total with the last dose being administered 3 months prior to his admission. On assessment, he had no symptoms of hypocalcaemia, such as perioral numbness and carpopedal spasm. He had no previous neck surgery or radiation and no significant family history of hypocalcaemia to suggest parathyroid gland injury or genetic disorder, respectively. He did not take any long-term supplements or complementary medicine. On physical examination, he had no neck scar with negative Chvostek’s and Trousseau’s sign and systemic review was unremarkable.

**Figure 1.**
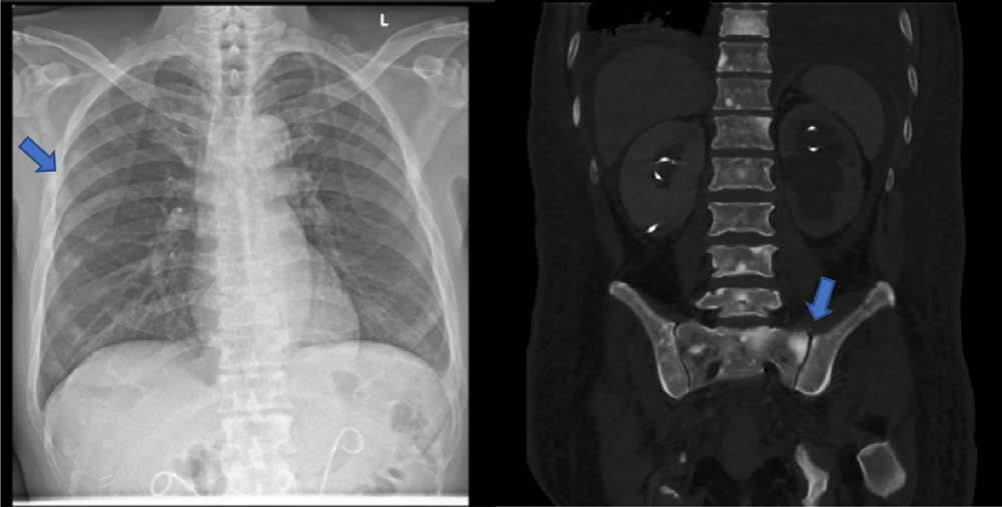
Chest radiography (CXR) and computed tomography (CT) scan showing extensive osteoblastic bone metastases (blue arrows).

On admission, the biochemical investigations revealed severe hypocalcaemia (corrected nonionized calcium 1.64 [2.09–2.46] mmol/L) with hypomagnesemia and hypophosphatemia. Intact parathyroid hormone (iPTH) level was markedly elevated in response to severe hypocalcaemia and vitamin D deficiency and that helped to exclude hypoparathyroidism (Table 1). He had no calcium measurement prior to admission for comparison. His electrocardiogram showed sinus tachycardia with no QTC prolongation. Chest radiography and computed tomography (CT) scan showed extensive areas of sclerosis in the bones suggestive of osteoblastic bone metastases (Fig. 1). Evaluation of the bone turnover markers demonstrated significant elevation of bone formation markers N-terminal propeptide of type I procollagen (P1NP) and alkaline phosphatase (ALP) at >18-fold and >13-fold respectively and comparative suppression of bone resorptive marker C-terminal telopeptide of type I collagen (CTX) which is indicative of a predominant osteoblastic activity resulting in disproportionate calcium influx into the bone (Table 1).

**Table 1:** Initial investigation on admission.

Investigations	Results (reference ranges)
eGFR	46 mL/min/1.73 m^2^
Nonionized calcium (corrected)	1.64 (2.09–2.46 mmol/L)
Phosphate	0.89 (0.94–1.50 mmol/L)
iPTH	49.2 (0.9–6.2 pmol/L)
Magnesium	0.62 (0.74–0.97 mmol/L)
25(OH)D	13.3 (10.6–111 ng/mL)
P1NP	1348 (18.4–72.3 UG/L)
Beta-cross lap	0.34 (<0.58 UG/L)

iPTH = intact parathyroid hormone, P1NP = N-terminal propeptide of type I procollagen.

**Figure 2.**
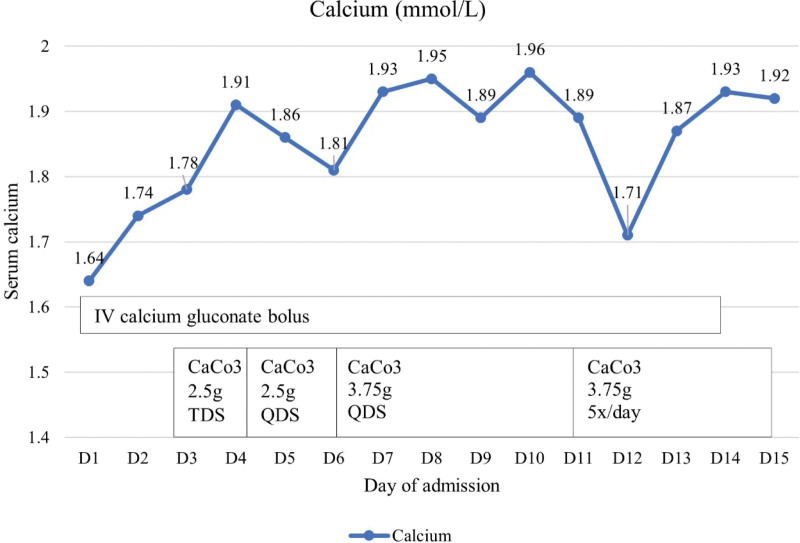
Calcium trend during the patient’s admission on calcium and vitamin D replacement.

His hypocalcaemia was contributed primarily by the underlying osteoblastic bone metastases and partially by the denosumab therapy, hypomagnesemia, and vitamin D deficiency. Denosumab was withheld and he was started on intravenous (IV) calcium gluconate boluses 2 to 5 cycles daily with concurrent oral replacement with calcium carbonate tablets 2.5 g 3 times daily, calcitriol 0.5 μg twice daily, cholecalciferol 50 000 IU once weekly and with magnesium replacement. Despite daily IV calcium replacement, oral calcium replacement with dose increment to 3.75 g 5 times daily, oral calcitriol replacement with dose increment to 1 μg 3 times daily, he had refractory and persistent hypocalcaemia with his calcium level ranging from 1.7 to 1.9 mmol/L (Fig. 2). He required a total of 16 days of IV calcium replacement with concurrent oral calcium and vitamin D replacement to maintain his corrected calcium at 1.92 mmol/L. He was discharged but was readmitted for symptomatic hypocalcaemia, symptomatic anaemia, fluid overload as well as pneumonia. He remained hypocalcaemic even after stopping denosumab for 6 months and he required high doses of calcium carbonate 1.25 g 4 times daily, calcitriol 1 μg 4 times daily, and cholecalciferol 25000 IU every 2 weekly to maintain his corrected calcium between 1.9 and 2.1 mmol/L. In view of the aggressive nature of the underlying metastatic bladder cancer and rapidly deteriorating functional status, he was deemed not a suitable candidate for systemic chemotherapy or radionuclide therapy. He was referred to the palliative team for comfort care and he eventually succumbed to the metastatic cancer 16 months after his diagnosis.

## 3. Discussion

Hypercalcemia is a common complication in malignancy, however, hypocalcaemia is less frequently reported and can occur in <2% of hospitalised patients.^[1]^ Hypocalcaemia in malignant disease can be secondary to various aetiologies categorised into nutritional deficiencies such as vitamin D deficiency, hypomagnesemia, malabsorption; secondary to treatment of the cancer or bone lesions such as chemotherapy or antiresorptive; or secondary to osteoblastic bone metastasis, hypoparathyroidism, tumour lysis syndrome, or acute illness.^[7]^

A comprehensive literature review of case reports on hypocalcaemia secondary to osteoblastic bone metastases published in year 1982 to 2020 were summarised in Table 2. A total of 21 cases were included and the age, gender, underlying primary malignancy, other contributing factors of hypocalcaemia and treatment modalities were reviewed. Prostate carcinoma was the most common malignancy with 12 cases followed by breast carcinoma (6 cases), gastric carcinoma (2 cases), and salivary gland carcinoma (1 case). The ages of the patients range from 37 to 88 years old. We found that in addition to osteoblastic bone metastases, concomitant vitamin D deficiency or relative hypoparathyroidism are common contributing factors of hypocalcaemia. We did not include patients on antiresorptive treatments as they were well-established contributors of refractory hypocalcaemia even in the absence of osteoblastic bone metastases.

**Table 2:** Summary for case reports of hypocalcaemia in osteoblastic bone metastases.

Year	Author	Age/gender	Cancer site/histology	Other contributing factors	Treatment
2006	Dawson et al^[5]^	70 yo/M	Salivary gland/undifferentiated	Relative hypoparathyroidism	Calcium and Vit D replacement
					Chemotherapy
					Radiotherapy
2017	Okazaki et al^[4]^	60 yo/F	Gastric/adenocarcinoma	Hx of total thyroidectomy	Calcium and Vit D replacement
					Chemotherapy
2020	Sakai et al^[8]^	87 yo/M	Gastric/signet ring cell	Nil	Not known
1982	Unger et al^[9]^	85 yo/F	Breast	Relative hypoparathyroidism	Not known
				Vit D deficiency	
1988	Hermus et al^[10]^	55 yo/F	Breast/poorly differentiated	Relative hypoparathyroidism	Calcium and Vit D replacement
1989	Bouvier et al^[11]^	39 yo/F	Breast	Relative hypoparathyroidism	Calcium and Vit D replacement
1994	Wiegand et al^[3]^	81 yo/F	Breast	Not known	Not known
2003	Bergkamp et al^[12]^	37 yo/F	Breast	Relative hypoparathyroidism	Calcium and Vit D replacement
					Chemotherapy
2015	Farolfi et al^[13]^	67 yo/F	Breast	Relative hypoparathyroidism	Calcium and Vit D replacement
				Vit D deficiency	
					Chemotherapy
					Hormonal treatment
1981	Smallridge et al^[14]^	61 yo/M	Prostate	Nil	Ca replacement
			Adenocarcinoma		
					Parathyroid extract
					Chemotherapy and hormonal treatment
1988	Kukreja et al^[15]^	88 yo/M	Prostate	Relative hypoparathyroidism	Calcium and Vit D replacement
			Adenocarcinoma	Vit D deficiency	Hormonal treatment
				CKD	
1995	Szentirmai et al^[16]^	82 yo/M	Prostate	Nil	Not known
			Adenocarcinoma		
2000	Lim et al^[17]^	80 yo/M	Prostate	Vit D resistance	Calcium and Vit D replacement
			Unknown		
2005	Fokkema at al^[18]^	67 yo/M	Prostate	Vit D deficiency	Calcium and Vit D replacement
			Adenocarcinoma	Acute kidney injury	Androgen deprivation therapy
2008	Yener et al^[19]^	70 yo/M	Prostate	Vit D deficiency	Calcium and Vit D replacement
			Adenocarcinoma		Androgen deprivation therapy
2008	Pusulari et al^[20]^	65 yo/M	Prostate	Nil	Not known
			Unknown		
2015	Rizzo et al^[21]^	72 yo/M	Prostate	Vit D deficiency	Calcium and Vit D replacement
			Unknown	Acute kidney injury	Androgen deprivation therapy
				Relative hypoparathyroidism	
2017	Gebara et al^[22]^	71 yo/M	Prostate	Vit D deficiency	Ca and Vit D replacement
			Adenocarcinoma		Steroid
2017	Diéguez et al^[23]^	84 yo/M	Prostate	Vit D deficiency	Calcium and Vit D replacement
			Adenocarcinoma		Androgen deprivation therapy
2018	Garla et al^[24]^	50 yo/M	Prostate	Vit D deficiency	Ca and Vit D replacement
			Unknown		Radium^223^ dichloride
2020	Drekolias et al^[25]^	61 yo/M	Prostate	Nil	Ca and Vit D replacement
			Adenocarcinoma		
					Chemotherapy and radiotherapy
					IV steroid

CKD = chronic kidney disease, Hx = history, IV = intravenous, vit D = vitamin D, yo = years old.

### 3.1. Risk factors contributing to the development of hypocalcaemia in malignancy

Hypocalcaemia secondary to osteoblastic bone metastases has been a well-established complication in prostate and breast cancer.^[2,3]^ It has been reported in gastric and salivary carcinoma but not in bladder cancer.^[4,5]^ Skeleton is one of the most frequent sites of metastasis after lung and liver^[26]^ and the relative incidence of bone metastasis in bladder cancer is 40%.^[6]^ Bone metastases increase the risks of morbidity and SREs such as hypercalcemia, bone pain, pathological fractures, cord compression, and bone marrow dysfunction and patients with bladder cancer and bone metastases generally have poorer prognosis with a median survival of 6 to 9 months.^[6]^ The bone metastasis found in bladder cancer can be classified as osteoblastic, osteolytic, or mixed.^[6]^ Metastasis altered bone homeostasis by decoupling the balance between bone resorption by osteoclasts and bone formation by osteoblasts.^[7,27]^ The type of bone metastasis depends on the activation and inhibition of osteoclasts and osteoblasts. Osteolytic bone metastasis is mediated by osteoclasts resulting in bone destruction and resorption. Major mediators of osteoclast activity include parathyroid hormone-related protein, bone-derived transforming growth factor-β, and receptor activator of nuclear factor-κB ligand.^[28,29]^ On the other hand, the exact pathogenesis of osteoblastic bone metastasis is still poorly elucidated. Osteoblastic bone lesions have been postulated to be stimulated by osteoblast growth factors such as platelet-derived growth factor, insulin-like growth factors, adrenomedullin, transforming growth factor, bone morphogenic proteins, and endothelin-1.^[6,29]^ The increased osteoblastic activity acts as a sink trap resulting in an increased uptake and utilisation of calcium leading to hypocalcaemia.

Classically, the biochemical investigations reveal low serum calcium, normal or low serum phosphate, low 25-hydroxyvitamin D, elevated iPTH, ALP, and other bone turnover markers.^[2]^ In terms of imaging, plain radiography, CT, magnetic resonance imaging (MRI), and bone scintigraphy are commonly utilised for the evaluation of bone metastases. Plain radiography is fast and inexpensive but has a low sensitivity of 44% to 50% while CT, MRI, and bone scintigraphy have better sensitivity of 74%, 95%, and 78%, respectively.^[6,27]^ Bone scintigraphy allows for evaluation of the whole skeleton and MRI is the imaging modality of choice for detection of metastatic lesion in the bone marrow as well as to assess for cord compression.^[27,30]^ In our patient, his biochemical abnormalities of hypocalcaemia, hypophosphatemia, raised iPTH and bone formation markers ALP and P1NP were typical of osteoblastic bone metastases. In addition, osteoblastic bone metastases were also reported on the chest radiography and CT imaging.

Not all patients with osteoblastic bone metastases develop hypocalcaemia and a previous study reported an incidence of at least 13% in patients with prostate cancer.^[31]^ Antiresorptive therapies such as bisphosphonates and denosumab which are traditionally used in the management of bone metastases are primarily osteoclast-targeted, with indirect and proportionately less effects on inhibition of osteoblasts.^[32]^ Antiresorptive agents have proven to be beneficial in lowering the risk of SREs,^[33,34]^ however, it can lead to severe and refractory hypocalcaemia especially in patients with chronic kidney disease, vitamin D deficiency, hypomagnesemia, or the presence of osteoblastic bone metastases.^[35–37]^ Patients with bone metastasis that is predominantly and aggressively osteoblastic have markedly increased risk of developing hypocalcaemia following antiresorptive treatment, as that further decouples the osteoclast-osteoblast activity. In the presence of hypocalcaemia, iPTH increases as a compensatory mechanism to increase osteoclastic bone resorption and thus maintaining normocalcaemia. The antiresorptive effect of denosumab or bisphosphonates inhibits this process resulting in unopposed sequestration of calcium into the skeleton leading to severe and protracted hypocalcaemia even with high doses of calcium replacement. Furthermore, it has been postulated that bisphosphonate at low doses might lead to osteoblast proliferation and thus the development of hypocalcaemia.^[38]^ Hypocalcaemia occurred more frequently (9.6%–13% in denosumab vs 3.9%–6% in zoledronic acid) and earlier in patients treated with denosumab compared to bisphosphonate such as zoledronic acid.^[36,38]^ The usage of vitamin D and calcium replacement has been shown to lower the risk of hypocalcaemia by 40%.^[36]^

Both hypomagnesemia and vitamin D deficiency can also contribute to the development of hypocalcaemia. Hypomagnesemia results in decreased PTH secretion as well as resistance to PTH action at the receptor.^[7,37]^ Vitamin D deficiency reduces gastrointestinal calcium absorption as well as kidney reabsorption of calcium.^[7,37]^ Vitamin D deficiency and hypomagnesemia is highly prevalent in patients with malignancy which can be secondary to medications and malnutrition. Based on the previously published case reports (Table 2), vitamin D deficiency is commonly observed in patients with hypocalcaemia in malignancy. In addition, vitamin D deficiency increases the risk of hypocalcaemia in patients treated with antiresorptive agent. As such, vitamin D and calcium levels should be routinely checked and replaced before the commencement of antiresorptive therapy which was not the case in our patient.

Relative hypoparathyroidism was found to be another factor that can aggravate hypocalcaemia. The proposed mechanisms of relative hypoparathyroidism were that of tissue destruction by tumour infiltration, radiation, postsurgical or secondary to autoimmune causes however no definite cause was found in most cases.^[9,11–13]^ Parathyroid metastases detected postmortem was reported in 6% to 12% of the oncologic patients.^[12]^ Interestingly, patients with breast cancer that developed hypocalcaemia were more likely to have relative hypoparathyroidism with inappropriately normal serum iPTH in relation to the severe hypocalcaemia, compared to patients with prostate cancer and it might be related to their history of radiation treatment (Table 2).

While the history of denosumab therapy, hypomagnesemia and vitamin D deficiency in our patient could contribute, in part, to the development of the hypocalcaemia, the clinical and biochemical features were, however, strongly suggestive of an osteoblastic process as the leading cause. Firstly, it is unusual for the hypocalcaemia to be evident only at 3 months after his 3rd dose of denosumab and to be persistent for 6 months after his last dose of denosumab. Denosumab has a mean half-life of 29 days and it suppresses the osteoclast activity maximally at approximately 2 weeks following its administration.^[37]^ Patients with prostate cancer treated with denosumab were reported to develop hypocalcaemia at a median of 16 days (range 4–35).^[37]^ Secondly, the pattern of his bone turnover markers was unlike that of typical postdenosumab effect, where both bone formation and resorption markers are expected to decrease.^[32]^ Instead, in our patient, the bone formation markers P1NP and ALP were significantly elevated, which was markedly discordant to the suppressed CTX. Therefore, profound uncoupling of bone remodelling processes due to direct tumoral effect should be considered and this disturbance of coupling depends on the interaction between the microenvironment and the bone. Tumour cell-derived factors in the bone environment are capable of inducing osteoblast-mediated bone formation as well as suppressing osteoclast-mediated bone resorption.^[39]^

### 3.2. Utility of bone turnover markers in bone metastases

In bone metastases, dysregulation of bone resorption and bone formation results in the generation of distinct biochemical markers which can be classified into bone formation markers and bone resorption markers. Bone formation markers commonly used in clinical and research setting include bone-specific ALP, osteocalcin, P1NP while bone resorption markers include pyridinoline, deoxypyridoline, aminoterminal crosslinked telopeptide of type I collagen, CTX, and carboxy-terminal crosslinked telopeptides of collagen type I.^[40]^ Bone turnover markers may provide insight into the state of bone remodelling, allow for prognostication of disease as well as evaluation of treatment response.^[40,41]^ Bone metastases stimulate bone remodelling and bone formation markers are elevated while bone resorption markers can be suppressed or elevated in osteoblastic metastases.^[39,40,42]^ The bone turnover profile in our patient reflected the dominant osteoblastic nature of the bone metastases. As such, these markers could be informative on the relative osteoblastic to osteoclastic activity in patients with sclerotic bone metastases. This may predict which patients will benefit from antiresorptive therapy or are at risk of treatment-induced hypocalcaemia, so that the choice of antiresorptive agent (denosumab versus bisphosphonate), calcium and vitamin D supplementation, as well as biochemical monitoring could be personalised to optimise treatment safety.

### 3.3. Treatment of hypocalcaemia in malignancy

There is no predefined guideline specific to the management of hypocalcaemia in osteoblastic bone metastases due to the rarity of this entity and initial treatment is similar to that of hypocalcaemia from other causes. In acute symptomatic hypocalcaemia, IV calcium gluconate 10 mL of 10% solution can be given as boluses followed by IV calcium infusion at a rate of 0.5 to 2 mg elemental calcium/kg/hour.^[43]^ Concomitant oral calcium, vitamin D, and calcitriol should be administered, and magnesium should be repleted. Frequent monitoring of symptoms, serial calcium measurements, and adjustment of the calcium and vitamin D replacements are the mainstay of management. However, in hypocalcaemia secondary to osteoblastic bone metastases, high doses of calcium, vitamin D, and calcitriol replacement are often needed as the hypocalcaemia is usually severe and refractory with some cases requiring prolonged IV calcium infusion.^[2,12]^ Treatment of the underlying cancer and bone metastases with chemotherapy have been shown to improve the hypocalcaemia and reduce the requirement of calcium and vitamin D supplementation.^[6,44]^ Radionuclide therapy such as radium^223^ dichloride and radioactive samarium used commonly for pain control due to bone metastasis has been described in case reports to normalise the serum calcium level.^[24,45]^ Glucocorticoid can result in suppression of bone formation through its action on the osteoclasts and osteoblasts.^[46]^ It has been used for the management of hypocalcaemia with mixed response.^[22,25]^ In our patient, he remained hypocalcaemic despite 2 weeks of IV calcium infusion in addition to high doses of calcium, vitamin D, and calcitriol replacement. Unfortunately, he was not a candidate for chemotherapy or radionuclide therapy due to his poor prognosis and functional status.

## 4. Conclusion

In conclusion, osteoblastic bone metastases lead to an increased influx of calcium and phosphate into the bone resulting in hypocalcaemia. Severe refractory hypocalcaemia is unusual even in patients with osteoblastic bone metastases and have been reported in prostate, breast, gastric and salivary cancer. To our knowledge, no cases of hypocalcaemia secondary to osteoblastic bone metastases in bladder cancer have ever been reported. This case illustrates the importance of recognising osteoblastic metastasis as a differential in protracted and severe hypocalcaemia in malignancy. It also highlights the importance of vitamin D repletion as well as serum calcium monitoring with the administration of antiresorptive agent especially in patients with osteoblastic bone metastases. Future study could evaluate the utility of bone turnover markers in predicting the benefits and risks of initiating antiresorptive agent in this group of patients.

## Author contributions

Conceptualization: Wanling Zeng, Du Soon Swee.

Data curation: Wanling Zeng, Du Soon Swee.

Writing—original draft: Wanling Zeng.

Writing—review and editing: Wanling Zeng, Du Soon Swee.

## References

[1] Blomqvist CP. A hospital survey of hypocalcemia in patients with malignant disease. Acta Med Scand. 2009;220:167–73. 10.1111/j.0954-6820.1986.tb02745.x 3776691

[2] Alfaro Riveros H Almodóvar LO Farriols Danés C. Hungry bone syndrome: persistent hypocalcemia related to osteoblastic bone metastases of prostate cancer. J Palliat Med. 2013;16:1496–7. 24160743 10.1089/jpm.2013.0389

[3] Wiegand MC Burshell A Jaspan J. Case report: clinical hypocalcemia: the Endocrine Conference of the Alton Ochsner Medical Institutions and Tulane University-Medical Center. Am J Med Sci. 1994;308:255–8. 7942987 10.1097/00000441-199430840-00009

[4] Okazaki J Muguruma N Kitamura S. Paraneoplastic hypocalcemia developed in Gastric cancer accompanied by Osteoblastic Metastasis. Intern Med. 2017;56:1345–9. 28566596 10.2169/internalmedicine.56.8545 PMC5498197

[5] Dawson SJ Murray RM Rischin D. Hypocalcemia associated with bone metastases in a patient with salivary-gland carcinoma. Nat Clin Pract Oncol. 2006;3:104–7. 16462851 10.1038/ncponc0405

[6] Macedo F Ladeira K Pinho F. Bone metastases: an overview. Oncol Rev 2017;11:321. 28584570 10.4081/oncol.2017.321 PMC5444408

[7] Schattner A Dubin I Huber R Gelber M. Hypocalcaemia of malignancy. Neth J Med. 2016;74:231–9. 27571720

[8] Sakai K Tomoda Y Saito H. Hungry bone syndrome and osteoblastic bone metastasis from gastric cancer. QJM Int J Med. 2020;113:903–904. 10.1093/qjmed/hcaa125 32298448

[9] Unger J Lignian H Brauman H. Hypocalcemia, osteoblastic metastases and hypoparathyroidism. Acta Clin Belg. 1982;37:247–9. 7148325 10.1080/22953337.1982.11718872

[10] Hermus A Beex L Liessum P. Hypocalcemia due to osteoblastic metastases and diminished parathyroid reserve in a patient with advanced breast cancer. Klin Wochenschr. 1988;66:643–6. 3210660 10.1007/BF01728807

[11] Bouvier DP. Hypocalcemia and an inappropriate endocrine response in osteoblastic metastatic breast cancer. South Med J. 1989;82:1574–6. 2556799 10.1097/00007611-198912000-00030

[12] Bergkamp FJM van Berkel AM van der Linden PWG Gorgels JPMC. Unexpected prolonged extreme hypocalcaemia and an inadequate PTH response in a patient with metastatic breast carcinoma. Neth J Med. 2003;61:371–5. 14768721

[13] Farolfi A Ferrario C Aquilina M. Paraneoplastic hypocalcemia-induced heart failure in advanced breast cancer: a case report and literature review. Oncol Lett 2015;10:773–7. 26622568 10.3892/ol.2015.3326 PMC4509413

[14] Smallridge RC Wray HL Schaaf M. Hypocalcemia with osteoblastic metastases in a patient with prostate carcinoma. Am J Med. 1981;71:184–8. 7246580 10.1016/0002-9343(81)90292-8

[15] Kukreja SC Shanmugam A Lad TE. Hypocalcemia in patients with prostate cancer. Calcif Tissue Int. 1988;43:340–5. 3146422 10.1007/BF02553276

[16] Szentirmai M Constantinou C Rainey JM. Hypocalcemia due to avid calcium uptake by osteoblastic metastases of prostate cancer. West J Med. 1995;163:577–8. 8553652 PMC1303276

[17] Lim SC Tan CE Aw TC. A man with osteoblastic metastasis and hypocalcaemia. Singapore Med J. 2000;41:74–6. 11063207

[18] Fokkema MI de Heide LJM van Schelven WD. Severe hypocalcaemia associated with extensive osteoblastic metastases in a patient with prostate cancer. Neth J Med. 2005;63:34–7. 15719851

[19] Yener S Demir O Ozdogan O. Severe hypocalcaemia because of osteoblastic prostate carcinoma metastases: Letters. Int J Clin Pract. 2008;62:1630–1. 18822033 10.1111/j.1742-1241.2008.01802.x

[20] Pusulari BB Akbar RA Butt M. Hypocalcemia with bony metastases in prostate cancer. J Ayub Med Coll Abbottabad JAMC. 2008;20:138–9. 19024209

[21] Rizzo C Vella S Cachia MJ. Refractory hypocalcaemia complicating metastatic prostatic carcinoma. BMJ Case Rep. 2015;2015:bcr2015210003. 10.1136/bcr-2015-210003 PMC4488614 26123464

[22] Gebara N Beaini C Karak FE. Refractory hypocalcemia effectively treated with steroids in prostate cancer metastatic to bone. Endocrinol Metab Syndr. 2017;6:269.

[23] Diéguez Felechosa M Noval Menéndez J Manjón Miguelez L. Severe hypocalcemia due to osteoblastic metastasis of prostate cancer. Med Clin (Barc). 2017;148:287–8. 28118964 10.1016/j.medcli.2016.12.021

[24] Garla VV Salim S Kovvuru KR. Hungry bone syndrome secondary to prostate cancer successfully treated with radium therapy. BMJ Case Rep. 2018;2018:bcr-2018-225039. 10.1136/bcr-2018-225039 PMC6040511 29982185

[25] Drekolias D Gonuguntla K Gadela NV. A rare case of severe sequestrational hypocalcemia in patient with metastatic prostate cancer. Chest. 2020;158:A842–3.

[26] Coleman RE. Metastatic bone disease: clinical features, pathophysiology and treatment strategies. Cancer Treat Rev. 2001;27:165–76. 11417967 10.1053/ctrv.2000.0210

[27] O’Sullivan GJ. Imaging of bone metastasis: an update. World J Radiol. 2015;7:202–11. 26339464 10.4329/wjr.v7.i8.202 PMC4553252

[28] Cecchini MG Wetterwald A van der Pluijm G. Molecular and biological mechanisms of bone metastasis. EAU Update Ser. 2005;3:214–226.

[29] Guise TA Mohammad KS Clines G. Basic mechanisms responsible for osteolytic and osteoblastic bone metastases: fig. 1. Clin Cancer Res. 2006;12:6213s–6s. 17062703 10.1158/1078-0432.CCR-06-1007

[30] Messiou C Cook G deSouza NM. Imaging metastatic bone disease from carcinoma of the prostate. Br J Cancer. 2009;101:1225–32. 19789531 10.1038/sj.bjc.6605334 PMC2768452

[31] Riancho JA Arjona R Valle R. The clinical spectrum of hypocalcaemia associated with bone metastases. J Intern Med. 1989;226:449–52. 2489231 10.1111/j.1365-2796.1989.tb01423.x

[32] Eastell R Christiansen C Grauer A. Effects of denosumab on bone turnover markers in postmenopausal osteoporosis. J Bone Miner Res. 2011;26:530–7. 20839290 10.1002/jbmr.251

[33] Saad F Gleason DM Murray R. Long-term efficacy of Zoledronic Acid for the prevention of skeletal complications in patients with metastatic hormone-refractory prostate cancer. JNCI J Natl Cancer Inst. 2004;96:879–82. 15173273 10.1093/jnci/djh141

[34] Fizazi K Carducci M Smith M. Denosumab versus zoledronic acid for treatment of bone metastases in men with castration-resistant prostate cancer: a randomised, double-blind study. Lancet. 2011;377:813–22. 21353695 10.1016/S0140-6736(10)62344-6 PMC3090685

[35] McCaleb RV Johnson JT. Severe, prolonged, denosumab-induced hypocalcemia with recovery after 111 days of high-dose calcium supplementation. AACE Clin Case Rep. 2019;5:e82–5. 31967007 10.4158/ACCR-2018-0295 PMC6876976

[36] Body JJ Bone HG de Boer RH. Hypocalcaemia in patients with metastatic bone disease treated with denosumab. Eur J Cancer. 2015;51:1812–21. 26093811 10.1016/j.ejca.2015.05.016

[37] Lau L Cliff ERS Wong V. Hypocalcaemia following denosumab in prostate cancer: a clinical review. Clin Endocrinol (Oxf). 2020;92:495–502. 32017154 10.1111/cen.14169

[38] Ho JW Sundar S. Prolonged hypocalcemia after Zoledronic Acid in a patient with metastatic prostate carcinoma: did Zoledronic Acid trigger osteoblastic activity and avid calcium uptake? Clin Genitourin Cancer. 2012;10:50–3. 22245101 10.1016/j.clgc.2011.11.004

[39] Clézardin P Coleman R Puppo M. Bone metastasis: mechanisms, therapies, and biomarkers. Physiol Rev. 2021;101:797–855. 33356915 10.1152/physrev.00012.2019

[40] Ferreira A Alho I Casimiro S. Bone remodeling markers and bone metastases: From cancer research to clinical implications. BoneKEy Rep. 2015;4:668. 25908969 10.1038/bonekey.2015.35 PMC4407509

[41] Wood SL Brown JE. Personal medicine and bone metastases: biomarkers, micro-RNAs and bone metastases. Cancers. 2020;12:2109. 10.3390/cancers12082109 PMC7465268 32751181

[42] de la Piedra C Alcaraz A Bellmunt J. Usefulness of bone turnover markers as predictors of mortality risk, disease progression and skeletal-related events appearance in patients with prostate cancer with bone metastases following treatment with zoledronic acid: TUGAMO study. Br J Cancer. 2013;108:2565–72. 23722472 10.1038/bjc.2013.270 PMC3694249

[43] Turner J Gittoes N Selby P. SOCIETY FOR ENDOCRINOLOGY ENDOCRINE EMERGENCY GUIDANCE: emergency management of acute hypocalcaemia in adult patients. Endocr Connect 2016;5:G7–8. 27935815 10.1530/EC-16-0056 PMC5314808

[44] Logothetis CJ Lin SH. Osteoblasts in prostate cancer metastasis to bone. Nat Rev Cancer. 2005;5:21–8. 15630412 10.1038/nrc1528

[45] Kassi E Kapsali I Kokkinos M. Treatment of severe hypocalcaemia due to osteoblastic metastases in a patient with post-thyroidectomy hypoparathyroidism with ^153^ Sm-EDTMP. BMJ Case Rep. 2017;2017:bcr-2017-219354. 10.1136/bcr-2017-219354 PMC5753734 28512123

[46] O’Brien CA Jia D Plotkin LI. Glucocorticoids act directly on osteoblasts and osteocytes to induce their apoptosis and reduce bone formation and strength. Endocrinology. 2004;145:1835–41. 14691012 10.1210/en.2003-0990

